# The Effects of Abstinence status and Sleep Quality on Resting State Alpha Power in Cocaine Use Disorder

**DOI:** 10.1177/15500594261450039

**Published:** 2026-05-11

**Authors:** Heather E. Webber, Danielle A. Kessler, Joy M. Schmitz, Scott D. Lane, Robert Suchting

**Affiliations:** 1 Faillace Department of Psychiatry and Behavioral Sciences, 12340 University of Texas Health Science Center at Houston, 1941 East Rd, Houston, TX, 77054, USA; 2 Department of Surgery, University of Colorado, 12631 East 17th Avenue, Room: 6111, Aurora, CO, 80045, USA

**Keywords:** cocaine, sleep, alpha power, resting-state EEG, substance use

## Abstract

**Rationale:**

Substance use disorders are associated with notable sleep disturbances. Electroencephalogram (EEG) alpha power occurs during wakeful rest, is associated with sleepiness, and indicates neural deactivation.

**Objectives:**

The goal of the current study was to assess the effects of abstinence on the relationship between alpha power and subjective sleep quality in participants with cocaine use disorder (CUD).

**Methods:**

Participants with CUD (n = 32) who were recently or non-abstinent completed a resting-state EEG paradigm (90 s eyes open/closed). Absolute alpha power was calculated using the fast Fourier transform and by averaging within the power spectra of the alpha band (8–13 Hz). The participants rated their subjective sleep quality on a scale of 1–10 for the past 7 nights. Bayesian linear mixed models evaluated the unique and moderating effects of sleep quality, group (abstinent, non-abstinent), eye condition (open, closed), and electrode location on resting-state alpha power.

**Results:**

Results indicated that subjective sleep quality did not differ between groups and that alpha power was decreased in the non-abstinent group. However, in the abstinent group only, better sleep quality was associated with decreased alpha power. There was no evidence of a relationship between sleep and alpha power in the non-abstinent group. This effect was similar across eye conditions, but was stronger at the right, posterior, and superior electrodes.

**Conclusions:**

These results could indicate a return to homeostasis during early abstinence or that those currently using might not get as much benefit from sleep, highlighting resting-state alpha power as a potential target for addressing sleep-related issues.

## Introduction

Substance use disorders are frequently accompanied by sleep disturbances, with cocaine use disorder (CUD) presenting particularly severe disruptions.^1,2^ Cocaine's stimulant properties and its modulation of neurochemical systems involved in sleep regulation contribute to these effects.^3,4^ In turn, poor sleep exacerbates core features of addiction, including impulsivity, craving, and relapse risk, while also impairing cognitive functions critical for recovery, such as attention and learning.^5–7^ Despite this bidirectional relationship, the neural mechanisms linking sleep quality and cocaine use—particularly across different stages of abstinence—remain poorly understood.

Resting-state electroencephalography (EEG) offers a noninvasive window into brain arousal states and has been used to characterize both sleep-related and substance-related neural dynamics. Alpha power (8–13 Hz), typically observed over occipito-parietal regions during relaxed wakefulness, is a well-established marker of cortical deactivation.^8^ It decreases during externally focused tasks and increases during rest or internally directed attention.^9–11^ Importantly, alpha power is sensitive to sleep pressure: it declines following sleep deprivation and is inversely related to subjective sleepiness and time awake.^12,13^ These dynamics are modulated by eye condition—alpha typically increases with eyes closed, but elevated alpha with eyes open may reflect disengagement and sleepiness.^14–17^

Cocaine use is also known to alter EEG rhythms. In rodents, acute administration of cocaine leads to a rapid decrease in alpha and increases in beta and gamma power.^18–20^ Human studies have yielded mixed findings, with some reporting increases in alpha and beta following acute use,^21,22^ while others observe beta changes.^23^ In contrast, chronic use and early abstinence are often associated with elevated resting-state alpha power,^24–27^ suggesting a potential rebound or withdrawal-related effect.

Despite these findings, the interaction between sleep quality and resting-state alpha power in individuals with CUD has not been systematically examined. This information is exigent, as sleep disturbances are a hallmark of cocaine withdrawal and improvements in sleep have been observed within a few weeks of abstinence.^2,28^ In a comprehensive review of sleep abnormalities in people who use cocaine, Angarita et al, found that the neurophysiological consequences of withdrawal from cocaine occur within the first few weeks of abstinence, with some measures resolving and some continuing after longer periods of abstinence.^29^ Specifically, sleep onset latency is increased during acute use, followed by a decrease during early abstinence. Total sleep time is highest within a few days of abstinence and decreases through the third week, but with over a month of abstinence, total sleep time may improve. Finally, slow wave sleep time is diminished after abstinence and may continue, while rapid eye movement sleep latency is decreased, but improves after 3 weeks of abstinence.^29^ Whether such improvements are reflected in neural markers of arousal, such as alpha power, remains unknown. Moreover, it is unclear whether the relationship between subjective sleep quality and alpha power differs between individuals who are non-abstinent versus those who are abstinent.

The present study addresses this gap by examining the relationship between subjective sleep quality and resting-state alpha power in individuals with CUD, comparing those who were non-abstinent to those who were recently abstinent (4 weeks to 6 months). This abstinence window was selected to minimize acute withdrawal effects as described above while still capturing early recovery to limit the heterogeneity of the sample. Participants completed a brief resting-state EEG protocol (eyes open, eyes closed) and rated their sleep quality over the past week. We hypothesized that (1) the abstinent group would report better sleep and exhibit lower alpha power relative to the non-abstinent group, and (2) that abstinence status would moderate the inverse relationship between sleep quality and alpha power, particularly in the eyes open condition, reflecting reduced sleep pressure and greater cortical engagement.

## Methods

### Participants

All participants (n = 32) were between the ages of 18–60 and met criteria for current moderate-to-severe cocaine use disorder (CUD) via the Structured Clinical Interview for DSM-5.^30^ Participants were excluded if they met for greater than mild substance use disorder for any illicit substance other than cocaine, were receiving treatment for psychiatric disorders or neurological diseases that would make participation unsafe, history of seizure disorders, or head injury with loss of consciousness in the past 5 years. For the non-abstinent group (n = 17), participants had to report cocaine use within the past 30 days or provide a urine drug screen that was positive for the cocaine metabolite benzoylecgonine during the intake process. For the abstinent group (n = 15), participants had to provide a urine drug screen that was negative for benzoylecgonine and report no cocaine use within the past 4 weeks and up to 6 months. The distribution of abstinence length is provided in the Table 1.

**Table 1.** Sample Characteristics.

Characteristic	Abstinent (n = 15)	Non-Abstinent (n = 17)
Age (M/SD)	50.21 (9.50)	52.54 (5.91)
Education (M/SD)	13.33 (1.63)	13.65 (1.62)
Sex (N Males)	11 (73.3%)	13 (76.47%)
Race (N African American)	10 (66.67%)	16 (94.12%)
Ethnicity (N Hispanic)	2 (13.33%)	0 (0%)
Years of Use (M/SD)	16.14 (12.80)	19 (9.48)
Days Since Use (M/SD)	64.87 (22.09)	3.53 (3.43)

### Procedures

Participants completed an in-person intake to determine eligibility. During this visit, they completed the Structured Clinical Interview for DSM-5, the Addiction Severity Index,^31^ and the Timeline Follow Back,^32^ and provided a urine sample for drug testing. If eligible, participants were scheduled for their study day. On the study day, the participants provided another urine sample and completed the Timeline Follow Back since last visit, the Cocaine Craving Questionnaire-brief, and the Sleep Quality Questionnaire. Following these assessments, the participants were fitted for an EEG cap and completed the resting state EEG measurements. This study was approved by the local IRB and all participants provided informed consent prior to participating in the study.

### Measures

*Sleep Quality*. Sleep quality was assessed with the single item sleep quality questionnaire.^33^ The SQS has established validity and test-retest reliability, reduces participant burden in clinical settings, and has been employed to assess sleep in addiction or mental health contexts.^34–38^ The participants are instructed to “Please think about the quality of your sleep overall, such as how many hours of sleep you got, how easily you fell asleep, you often you woke up during the night (except to go to the bathroom), how often you woke up earlier than you had to in the morning, and how refreshing your sleep was.” Then the participants are asked to rate their sleep quality overall on a scale of 0 to 10 in 1-point increments (terrible to excellent). Participants completed the scale two times, once thinking about the past 7 days and once thinking only about the last night. Past 7 days is a stronger representation of overall recent sleep and was used in the primary analyses. For analyses involving last night sleep, please see the Supplemental Materials.

*Resting State Alpha*. EEG was recorded for a total of 3 min while the participants were sitting up, awake, in a chair in a lit room. For the eyes opened condition, participants were instructed to sit still and look at the cross presented on the screen and blink normally for 90 s. For the eyes closed condition, participants were instructed to sit still and close their eyes for 90 s. EEG was acquired with a 64-channel actiCAP, amplified with Brain Vision BrainAmp MR, and digitized with Brain Vision Recorder (Brain Products, Munich, Germany). Data were referenced to FCz, sampled at a rate of 500 Hz and filtered online (.1–100 Hz).

### Data Analysis

*EEG Data Analysis*. Data were re-referenced to the average reference. Eye blinks and movements were corrected using Independent Components Analysis. Data were cut into 2-s segments in order to produce stationarity, have an optimal frequency resolution, and for artifact detection and removal consistent with prior work.^12,39,40^ Data were inspected for artifacts and channels that were contaminated by artifacts in more than 40% of the segments were interpolated using Hjorth Laplacian Method.^41^ If a segment had more than 10% of the sensors with artifacts, these segments were marked bad and not included in the average power analysis. On average, 12 independent components were rejected and 11% of segments were removed, leaving around 80 s of artifact-free resting EEG data per condition. Absolute alpha power was estimated using the Fast Fourier Transform on the cleaned 2-s segments using a 10% Hanning window. Power spectra data were log-transformed before average power was calculated. Alpha power was defined as the average power between 8–13 Hz at each electrode and was calculated separately for the eyes opened and eyes closed conditions. A split half analysis was conducted to assess the stability of alpha over the course of the 90 s segment and to show internal consistency of the metric. Pearson correlations between alpha power during the first half and second half of the recording were performed at each electrode separately for both conditions. Results indicated acceptable (> *r* = .7) consistency for both conditions at all electrodes with the exception of 3 electrodes (FC3, CP2, P04) which showed (> r = .67).

*Statistical Analyses*. Descriptive statistics were used to characterize the sample (eg, mean ± SD for continuous variables; n/% for categorical variables). Preliminary modeling tested potential differences between cocaine use group (abstinent vs non-abstinent; hereafter “group”) with respect to sleep. Primary analyses focused on the interactive effects of group and sleep (average sleep quality over the previous week) on alpha power measured across 65 electrodes under two eye conditions (closed/open). Alpha power measurements were transposed to provide 130 rows per participant (65 electrodes x 2 eye conditions). Each electrode was encoded along three Cartesian axes by converting spherical coordinates (theta, phi) originally specified for each electrode in the actiCAP: medial-lateral (ML; x-axis, left-to-right, sagittal plane), anterior-posterior (AP; y-axis, back-to-front, coronal plane), and inferior-superior (IS; z-axis, bottom-to-top, transverse plane). This continuous spatial encoding preserves the full topographic geometry of the electrode array and avoids information loss associated with discretization into regional clusters.

Bayesian linear mixed models (LMM) evaluated the unique and moderating effects of sleep, group, eye condition, and electrode location. Models 1–2 evaluated the bivariate effects of group and sleep, respectively, controlling for eye condition and electrode location. Model 3 provided the primary model, evaluating the potential interaction between sleep and group (abstinent vs non-abstinent), adjusted for eye condition, electrode location, and main effects of sleep and group. Models 4–7 evaluated the potential moderating influence of eye condition and electrode location on the interaction between sleep and cocaine use group. Fixed effects for each model are transcribed below, where interactions include all non-redundant constituent lower-order effects.

Model 1: alpha power ∼ Group + Eyes + ML x AP x IS
Model 2: alpha power ∼ Sleep + Eyes + ML x AP x IS
Model 3: alpha power ∼ Sleep x Group + Eyes + ML x AP x IS
Model 4: alpha power ∼ Sleep x Group x Eyes + ML x AP x IS
Model 5: alpha power ∼ Sleep x Group x ML + Eyes + ML x AP x IS
Model 6: alpha power ∼ Sleep x Group x AP + Eyes + ML x AP x IS
Model 7: alpha power ∼ Sleep x Group x IS + Eyes + ML x AP x IS

Each LMM included a random intercept term for participant to accommodate within-subject correlation. Multilevel modeling enabled partial pooling across electrodes and eye conditions, stabilizing estimates despite the modest sample size. Weakly informative priors (*b* ∼ *N*(*µ* = 0; *σ* = 10)) introduced mild shrinkage toward zero to guide against extreme coefficients. Convergence and fit were assessed via R-hat, effective sample size, and posterior predictive checks.

The Bayesian framework was preferred for its ability to directly quantify the probability that model effects exist, given weakly informative priors (*b* ∼ *N*(*µ* = 0; *σ* = 10)) and the observed data. Bayesian inference was chosen over traditional frequentist inference to leverage this accessible conceptualization of probability rather than relying on null hypothesis statistical testing and p-values. Further, Bayesian estimation with weakly informative priors provides more stable parameter estimates in complex multilevel models with many parameters, particularly when modeling continuous spatial coordinates across 65 electrodes.^42^ Results are reported as the posterior median, 95% credible interval (CrI), and posterior probability (**PP**) that a coefficient differs from zero (ie, the proportion of the posterior distribution that was greater or less than the null effect). Heuristics from the literature^43–45^ were used to characterize degrees of evidence in the following strata: *none* (PP = 50%), *anecdotal* (PP = 51%–74%), *moderate* (PP = 75%–90%), *strong* (PP = 91%–96%), *very strong* (PP = 97%–99%), and *extreme* (PP > 99%). Analyses were conducted in R v. 4.5.0.^46^ via packages *brms* v. 2.22.0^47^ and *marginaleffects* v. 0.25.1.^48^

## Results

### Sample Characteristics

Participants were on average 51 years old, 25% female, 81% Black/African American, with 13 years of education. Participants used cocaine on average 6.8 days of the past 30 days and had been using for 17.7 years. All participants in the abstinent group tested negative for benzoylecgonine at the intake and on the day of the assessment and reported not using for a range of 30–104 days. 15/17 participants in the non-abstinent group tested positive the day of the EEG and reported not using for an average of 3.5 days (range 1–13) prior to the appointment. Full sample characteristics by group are presented in Table 1.

### Bayesian Linear Mixed Models

**Figure 1.**
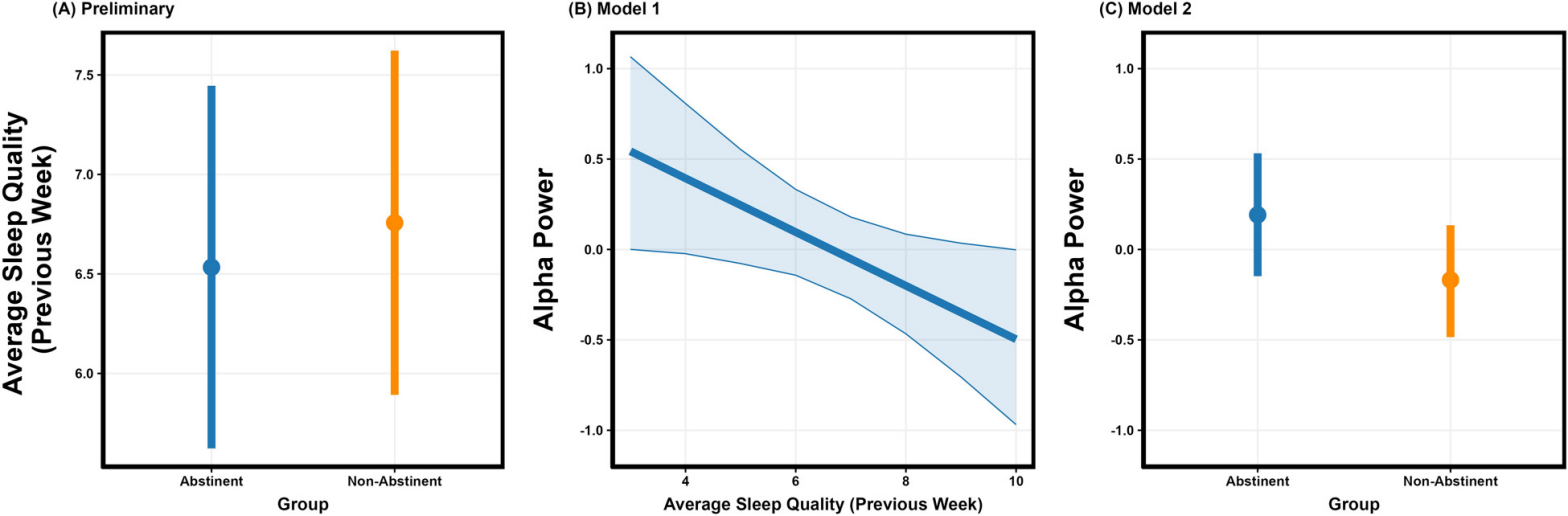
Preliminary model and main effects (models 1–2) a) Sleep quality by group b) Relationship between alpha power and sleep quality c) Alpha power by group.

Seven Bayesian LMMs were fit to examine the independent and interactive effects of sleep, cocaine use group (abstinent vs non-abstinent), eye condition (closed/open), and electrode position on alpha power. Posterior medians, 95% CrIs, and PPs for each interaction effect are described in text below; marginal slopes for each group are described broadly and provided in full in Table 2. Before running the primary models described below, a preliminary model fit sleep quality as a function of group (Figure 1a). This model did not find support for different sleep quality across groups (*b* = 0.23, 95% CrI [−1.03, 1.50], PP = 64.1%), and similar results were obtained when examining raw abstinence duration in place of group (PP = 66.9%).

**Table 2.** Marginalized Slopes and Credible Interval (CrI) for the Effect of Sleep on Alpha Power Within Cocaine Use Group and Moderating Strata.

**Model**	**Moderator**	**Group**	**Slope [95% CrI]**	**PP**
**3**	N/A	Abstinent	−0.19 [−0.35,−0.37]	99.0%
		Non-Abstinent	−0.05 [−0.26, 0.16]	66.7%
**4**	Closed	Abstinent	−0.19 [−0.35,−0.03]	99.0%
	Open	Abstinent	−0.20 [−0.37,−0.04]	99.1%
	Closed	Non-Abstinent	−0.06 [−0.27, 0.16]	70.8%
	Open	Non-Abstinent	−0.02 [−0.23, 0.19]	59.3%
**5**	Left	Abstinent	−0.17 [−0.33,−0.01]	98.2%
	Midline	Abstinent	−0.20 [−0.35,−0.04]	99.3%
	Right	Abstinent	−0.22 [−0.37,−0.06]	99.6%
	Left	Non-Abstinent	0.01 [−0.20, 0.22]	53.1%
	Midline	Non-Abstinent	−0.04 [−0.25, 0.17]	65.3%
	Right	Non-Abstinent	−0.10 [−0.30, 0.12]	81.4%
**6**	Posterior	Abstinent	−0.25 [−0.41,−0.09]	99.9%
	Central	Abstinent	−0.20 [−0.35,−0.04]	99.1%
	Anterior	Abstinent	−0.14 [−0.30, 0.02]	95.6%
	Posterior	Non-Abstinent	−0.06 [−0.26, 0.16]	70.8%
	Central	Non-Abstinent	−0.04 [−0.25, 0.17]	66.1%
	Anterior	Non-Abstinent	−0.03 [−0.24, 0.19]	60.7%
**7**	Inferior	Abstinent	−0.16 [−0.32, 0.01]	97.3%
	Mid-Transverse	Abstinent	−0.20 [−0.36,−0.04]	99.7%
	Superior	Abstinent	−0.24 [−0.40,−0.08]	99.7%
	Inferior	Non-Abstinent	0.04 [−0.25, 0.17]	64.3%
	Mid-Transverse	Non-Abstinent	−0.04 [−0.25, 0.17]	65.3%
	Superior	Non-Abstinent	0.05 [−0.25, 0.17]	66.2%

**Models 1–2 (Sleep and Group Main Effects).** Each one-point increase in sleep quality was associated with lower alpha power (*b* = −0.15, 95% CrI [−0.28, −0.02], PP = 98.6%) (Figure 1b), for a total difference of Δ*µV*^2^ = −1.02 across the observed range of sleep quality in the data. The non-abstinent group demonstrated lower alpha power than the abstinent group (*b* = −0.36, [−0.82, 0.10], PP = 94.1%) (Figure 1c). Median alpha power was positive in the abstinent group (0.20, [−0.48, 0.14], PP = 87.4%) and negative in the non-abstinent group (−0.16, [−0.48, 0.14], PP = 86.5%).

**Figure 2.**
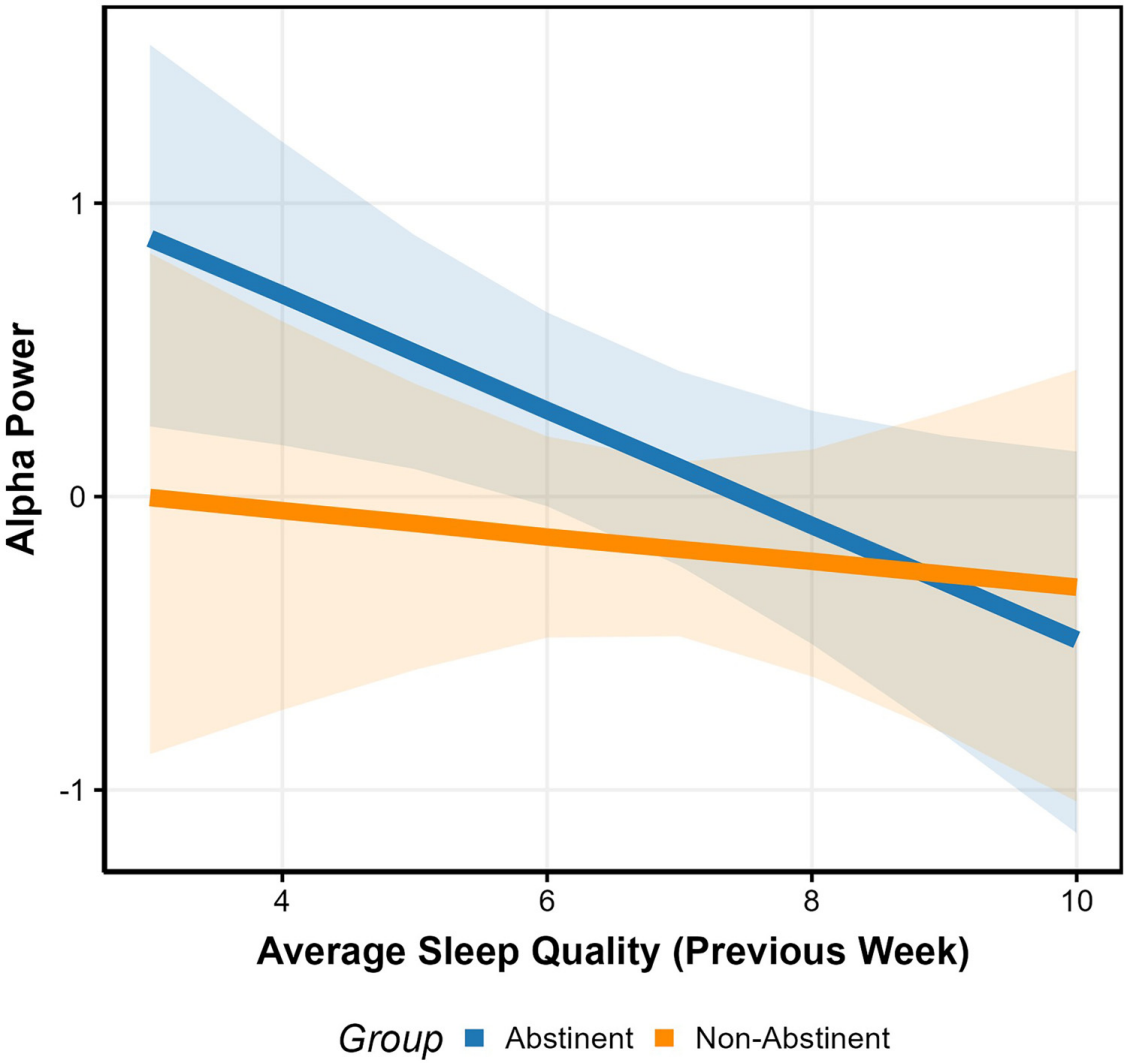
Sleep by group interaction (model 3).

**Model 3 (Sleep x Group).** The effect of sleep on alpha power differed by group (sleep x group: *b* = 0.16, 95% CrI [−0.11, 0.42], PP = 87.8%). In the abstinent group, alpha power declined with higher sleep quality (Δ*µV*^2^ = −1.37, slope = −0.19, [−0.35, −0.03], PP = 99.0%), while the non-abstinent group demonstrated no relationship between alpha power and sleep quality (Δ*µV*^2^ = −0.31, slope = −0.05, [−0.26, 0.16], 66.7%) (Figure 2).

**Figure 3.**
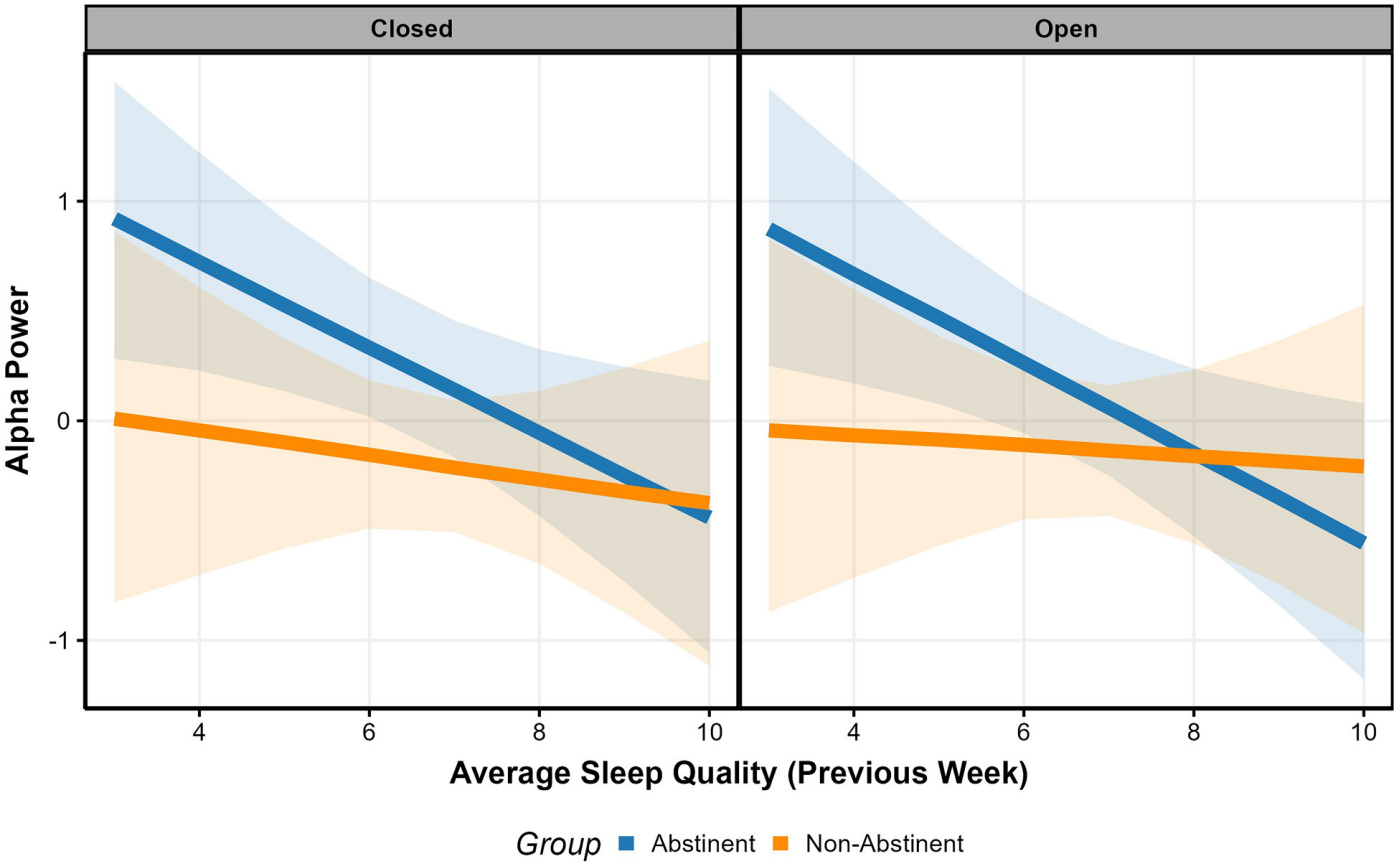
Sleep by group by eyes interaction (model 4).

**Model 4 (Sleep x Group x Eyes).** The three-way interaction was supported (*b* = 0.04, 95% CrI [−0.01, 0.10], PP = 92.9%). As above, sleep quality was negatively related to alpha power in the abstinent group whether eyes were closed (Δ*µV*^2^ = −1.36, slope = −0.19, [−0.35, −0.03], PP = 99.0%) or open (Δ*µV*^2^ = −1.43, slope = −0.20, [−0.37, −0.04], PP = 99.1%). In the non-abstinent group, the association between sleep and alpha power was not supported whether eyes were closed or open (PP < 75%) (Figure 3).

**Models 5–7 (Sleep x Group x Electrode Axis [ML/AP/IS]).** Each axis-specific three-way interaction was supported (ML: PP = 97.3%; AP: PP = 99.8%; IS: PP = 99.6%). For the non-abstinent group, sleep showed insufficient evidence for an association with alpha power across regional strata (PP < 75), except for a modest negative slope in the right-hemisphere ML stratum (Δ*µV*^2^ = −0.69, slope = −0.10, PP = 81.4%). Conversely, sleep in the abstinent group exhibited strong to extreme evidence (PP = 95.6–99.9%) for an association with alpha power in every stratum, with effect sizes increasing when viewed from left to right (−0.17 to −0.22; Δ*µV*^2^ = −1.21 to −1.55), anterior to posterior (−0.14 to −0.25; Δ*µV*^2^ = −0.99 to −1.76,), and inferior to superior (−0.16 to −0.24; Δ*µV*^2^ = −1.11 to −1.69). Figures of these interactions are presented in Supplemental Figures 7–9. Finally, exploratory models were fit to evaluate the potential influence of eye condition on the three-way interactions described by Models 5–7. These models explored the higher-order 4-way interaction between eye condition, abstinence group, sleep quality, and electrode axis location. The models focusing on the medial-lateral and inferior-superior axes demonstrated anecdotal support (PP < 75%); however, the higher order interaction was supported for the model focusing on the anterior-posterior axis. Tests of marginal effects found support for the sleep x group x AP axis interaction within both conditions (Eyes Open: *b* = −0.352, PP = 93.3%; Eyes Closed: *b* = −0.247, PP = 85.1%) and strong support for the contrast between conditions (*b* = −0.106, PP = 90.5%), indicating that although the effect was supported in both conditions, and in the same direction, it was stronger for the Open condition.

## Discussion

The present study examined the relationship between subjective sleep quality and resting-state alpha power in individuals with CUD, comparing those currently using cocaine to those in early abstinence. Contrary to our initial hypothesis, subjective sleep quality did not differ between groups. However, alpha power was elevated in the abstinent group, and critically, a negative association between sleep quality and alpha power emerged only in this group. These findings offer novel insights into the neurophysiological correlates of sleep quality during early recovery from cocaine use.

Prior work has indicated that alpha power is increased within 5 days of abstinence and during an extended withdrawal period.^24–26^ We similarly found that with at least four weeks of abstinence, alpha power is increased compared to those with less than 4 weeks of abstinence. The non-abstinent group reported here was assessed on average 3.5 days since their last use. It is possible that the non-abstinent group still has elevated alpha power in comparison to when they were actively using (or in comparison to a healthy control group), but that data was not collected as a part of this project. Further, prior studies have only assessed these effects up to 30 min to a few hours after controlled cocaine administration.^21,22^ An interesting avenue for future work would be to assess the direct time course of when alpha increases after use.

There are several functional interpretations of alpha reported within the literature. Classic posterior alpha power is typically interpreted as functional inhibition and disengagement of task-irrelevant cortical regions and is reliably suppressed with visual input.^49^ In contrast, intrusion of alpha activity during the eyes opened condition has been associated with reduced vigilance or hypoarousal states.^50,51^ Here, we found that in abstinent individuals with CUD, poorer sleep quality was associated with increased alpha power across both eye conditions, with stronger effects for the eyes opened condition. This is largely consistent with the literature describing drowsiness or reduced vigilance during eyes opened after poor sleep.^17^ This pattern suggests that even brief periods of quiet rest may elicit rapid neural deactivation in the context of suboptimal sleep, potentially reflecting heightened sleep pressure, mental fatigue, and difficulty maintaining alertness.^52,53^ Therefore, increased alpha in clinical populations affected by altered arousal states, such as those with substance use disorders, may indicate sleep effects on maintaining arousal.^54^ While these results may reflect alpha intrusion rather than classic posterior alpha rhythms, it is also possible that the brevity of the EEG recording (90 s per condition) may have limited the emergence of condition-specific effects, such as alpha dropout during eyes-closed rest. Finally, in addition to sleep pressure or maintaining alertness, the increased alpha observed with poorer sleep in abstinent individuals could reflect a cortical inhibition or “gating” mechanism to ignore irrelevant sensory information.^55^ Future work could assess alpha power under different cognitive conditions rather than simply at rest to further parse the functional significance of these findings.

A central finding of this study is that the relationship between sleep quality and alpha power was contingent on cocaine use status. In abstinent individuals, better sleep quality predicted lower alpha power, consistent with reduced sleep pressure and greater cortical engagement with external stimuli. In contrast, no such relationship was observed in the non-abstinent group. This dissociation may reflect a partial restoration of homeostatic regulation in early abstinence, whereby the brain becomes more responsive to variations in sleep quality. Alternatively, non-abstinent individuals may experience a blunted neurophysiological response to sleep due to ongoing neurochemical dysregulation or may lack insight into their sleep quality—a phenomenon well-documented in substance-using populations.^2^ These interpretations underscore the importance of integrating both subjective and objective sleep measures in future research.

The continuous spatial electrode coordinate analysis allowed for a unique view of the findings with regards to the spatial distribution of alpha. The spatial distribution of the sleep–alpha association revealed a consistent pattern: effects were strongest at right-lateralized, posterior/occipital, and superior electrode sites. As the results were strongest at the back of the head, the results can be contextualized within the posterior alpha literature, which has been implicated in arousal and vigilance networks and is interpreted as a marker of cortical idling and arousal regulation.^56^ The posterior dominance observed here aligns with models linking poor sleep to impaired regulation of vigilance and mental fatigue, rather than to more affective processes typically associated with frontal alpha asymmetry.^57^ Although the present analyses were conducted at the scalp level and are influence by volume conduction, the topography is broadly consistent with prior combined EEG-fMRI work that suggests occipital sources and broader areas involved in arousal and vigilance such as the default mode network.^58–62^ Further, the right-lateralized alpha effects are consistent with a large body of literature indicating the right lateralization of functional networks involved in arousal and vigilance.^63–65^ Finally, the right lateralization here may also reflect a shift toward withdrawal-related motivational states, as reduced left relative to right alpha has been associated with avoidance behavior.^66^ Thus, poor sleep in abstinent individuals may not only impair arousal regulation but also bias motivational systems in ways that could impact recovery trajectories.

This study leveraged a brief, clinically feasible EEG protocol and a validated single-item sleep measure to assess neurophysiological correlates of sleep in CUD. The use of Bayesian linear mixed models allowed for robust estimation of effects across a high-dimensional electrode space, addressing common limitations in EEG research such as multiple comparisons and electrode selection bias. However, several limitations warrant consideration. First, the modest sample size, particularly in the non-abstinent group (n = 17), may have resulted in less precise estimates for this subgroup, as reflected in the wider credible intervals. Although the Bayesian methods used here do not depend on conventional statistical power, replication with a larger and/or more balanced sample would improve support in the group-specific patterns observed in the present analyses. Second, while the single-item sleep measure reduced burden and required minimal cognitive ability, these results should be replicated with more robust, multi-item tools such as the Pittsburg Sleep Quality Index. Third, the absence of objective sleep measures (eg, actigraphy or polysomnography) limits the interpretability of subjective reports, especially given known discrepancies in SUD populations. Fourth, the short EEG recording may have precluded detection of dynamic changes in alpha power over time or across eye conditions. Finally, while not the main aim of the current study, the lack of normative data or a healthy control comparison group limited our ability to conclude whether alpha power levels observed here are indicative of disease state. Future work would benefit from identifying how the groups differ from healthy controls in their alpha power levels and alpha power's association with sleep quality.

Together, these findings suggest that resting-state alpha power may serve as a sensitive marker of sleep-related neural deactivation and reduced vigilance in individuals recovering from cocaine use. The absence of this relationship in the non-abstinent group highlights the potential utility of alpha power as an index of neurophysiological recovery and a candidate biomarker for treatment monitoring. These results also raise the possibility that alpha may reflect a neurophysiological recovery process and future longitudinal studies could examine how the alpha-sleep quality relationship evolves over CUD recovery stages. In addition to serving as a marker for treatment change, alpha alterations could also be directly targeted via neurofeedback, a potentially efficacious intervention that has been tested in substance use disorders.^67,68^ Interestingly, a few studies have also applied neurofeedback to improve sleep quality, with minimal evidence of an effect.^69^ Future work may seek to consider both cocaine use and sleep quality effects on resting-state dynamics to develop novel parameters for feedback. Finally, future studies should incorporate multimodal sleep assessments and longitudinal designs to clarify the temporal dynamics of these relationship and their relevance for clinical outcomes. If validated, alpha power could inform targeted interventions aimed at mitigating sleep-related cognitive and affective vulnerabilities in CUD.

## Supplemental Material

sj-docx-1-eeg-10.1177_15500594261450039 - Supplemental material for The Effects of Abstinence status and Sleep Quality on Resting State Alpha Power in Cocaine Use Disorder Supplemental material, sj-docx-1-eeg-10.1177_15500594261450039 for The Effects of Abstinence status and Sleep Quality on Resting State Alpha Power in Cocaine Use Disorder by Heather E. Webber, Danielle A. Kessler, Joy M. Schmitz, Scott D. Lane and Robert Suchting in Clinical EEG and Neuroscience
