# Supplementary material for: The Effects of Abstinence status and Sleep Quality on Resting State Alpha Power in Cocaine Use Disorder
Source: Clin EEG Neurosci. 2026 May 11;57(4):341–50. doi: 10.1177/15500594261450039 (PMC13237756; doi:10.1177/15500594261450039)
Supplement: sj-docx-1-eeg-10.1177_15500594261450039 - Supplemental material for The Effects of Abstinence status and Sleep Quality on Resting State Alpha Power in Cocaine Use Disorder [file sj-docx-1-eeg-10.1177_15500594261450039.docx]

**Supplementary Material for:**

The effects of abstinence status and sleep quality on resting state alpha power in cocaine use disorder

**Supplementary Material**

**Secondary Analyses – Overview**

Details for the secondary analyses follow from those reported for the primary analyses, with the main change being that the primary analyses operationalized sleep quality over the previous seven days, whereas the secondary analyses explored sleep quality from the previous night only. Some analytic details described in the manuscript are reiterated here for convenience. Preliminary modeling tested potential differences between cocaine use group (abstinent vs. non-abstinent; hereafter “group”) with respect to sleep. Primary analyses focused on the interactive effects of group and sleep *(sleep quality on the previous night*) on alpha power measured across 65 electrodes under two eye conditions (closed/open). Alpha power measurements were transposed to provide 130 rows per participant (65 electrodes x 2 eye conditions). Each electrode was encoded along three Cartesian axes: medial-lateral (ML; x-axis, left-to-right, sagittal plane), anterior-posterior (AP; y-axis, back-to-front, coronal plane), and inferior-superior (IS; z-axis, bottom-to-top, transverse plane).

Bayesian linear mixed models (LMM) evaluated the unique and moderating effects of sleep, group, eye condition, and electrode location. Models S1-S2 evaluated the bivariate effects of group and sleep, respectively, controlling for eye condition and electrode location. Model S3 provided the primary model, evaluating the potential interaction between sleep and group (abstinent vs. non-abstinent), adjusted for eye condition, electrode location, and main effects of sleep and group. Models S4-S7 evaluated the potential moderating influence of eye condition and electrode location on the interaction between sleep and cocaine use group. Fixed effects for each model are transcribed below, where interactions include all non-redundant constituent lower-order effects.

Model S1: alpha power ~ Group + Eyes + ML x AP x IS

Model S2: alpha power ~ Sleep + Eyes + ML x AP x IS

Model S3: alpha power ~ Sleep x Group + Eyes + ML x AP x IS

Model S4: alpha power ~ Sleep x Group x Eyes + ML x AP x IS

Model S5: alpha power ~ Sleep x Group x ML + Eyes + ML x AP x IS

Model S6: alpha power ~ Sleep x Group x AP + Eyes + ML x AP x IS

Model S7: alpha power ~ Sleep x Group x IS + Eyes + ML x AP x IS

Each LMM included a random intercept term for participant to accommodate within-subject correlation. Multilevel modeling enabled partial pooling across electrodes and eye conditions, stabilizing estimates despite the modest sample size. Weakly informative priors (*b* ~ *N*(*µ* = 0; *σ* = 10)) introduced mild shrinkage toward zero to guide against extreme coefficients. Convergence and fit were assessed via R-hat, effective sample size, and posterior predictive checks. The Bayesian framework was preferred for its ability to directly quantify the probability that model effects exist, given weakly informative priors (*b* ~ *N*(*µ* = 0; *σ* = 10)) and the observed data. Results are reported as the posterior median, 95% credible interval (CrI), and posterior probability (**PP**) that a coefficient differs from zero (i.e., the proportion of the posterior distribution that was greater or less than the null effect). Heuristics from the literature (Andraszewicz et al., 2015; Jeffreys, 2006; Lee & Wagenmakers, 2013) were used to characterize degrees of evidence in the following strata: *none* (PP=50%), *anecdotal* (PP = 51-74%), *moderate* (PP=75-90%), *strong* (PP=91-96%), *very strong* (PP=97-99%), and *extreme* (PP > 99%).

**Secondary Analyses - Results**

Posterior medians, 95% CrIs, and PPs for each interaction effect are described in text below; marginal slopes for each group are described broadly and provided in full in Table S1. Before running the primary models described below, a preliminary model fit sleep quality as a function of group. This model supported different previous night sleep quality across groups (*b* = 1.48, 95% CrI [-0.15, 3.11], PP = 96.3%) (Figure S1A). This difference from the primary analyses did not appear to have cascading effects in downstream analyses with respect to the sleep x group and higher-order interactions. The marginal slopes and posterior probabilities describing the last night sleep quality-alpha power relationship with subgroup strata were largely the same, but blunted, relative to the primary analyses (with previous week average sleep quality).

**Table S1. Marginal slope within grouping strata by model.**

| **Model** | **Moderator** | **Group** | **Slope [95% CrI]** | **PP** |
| --- | --- | --- | --- | --- |
| **S3** | N/A | Abstinent | -0.10 [-0.22, 0.03] | 93.9% |
|  |  | Non-Abstinent | -0.04 [-0.23, 0.16] | 64.2% |
| **S4** | Closed | Abstinent | -0.09 [-0.21, 0.04] | 92.2% |
|  | Open | Abstinent | -0.11 [-0.23, 0.02] | 94.9% |
|  | Closed | Non-Abstinent | -0.07 [-0.27, 0.13] | 77.5% |
|  | Open | Non-Abstinent | -0.01 [-0.20, 0.20] | 51.5% |
| **S5** | Left | Abstinent | -0.07 [-0.20, 0.05] | 87.9% |
|  | Midline | Abstinent | -0.10 [-0.23, 0.03] | 94.3% |
|  | Right | Abstinent | -0.13 [-0.25,-0.01] | 97.7% |
|  | Left | Non-Abstinent | -0.03 [-0.23, 0.17] | 60.9% |
|  | Midline | Non-Abstinent | -0.04 [-0.24, 0.15] | 66.0% |
|  | Right | Non-Abstinent | -0.05 [-0.25, 0.14] | 70.6% |
| **S6** | Posterior | Abstinent | -0.13 [-0.26,-0.01] | 98.3% |
|  | Central | Abstinent | -0.10 [-0.22, 0.03] | 94.5% |
|  | Anterior | Abstinent | -0.06 [-0.19, 0.06] | 84.1% |
|  | Posterior | Non-Abstinent | -0.05 [-0.25, 0.14] | 70.8% |
|  | Central | Non-Abstinent | -0.04 [-0.23, 0.16] | 65.6% |
|  | Anterior | Non-Abstinent | -0.02 [-0.22, 0.17] | 59.3% |
| **S7** | Inferior | Abstinent | -0.07 [-0.20, 0.05] | 87.6% |
|  | Mid-Transverse | Abstinent | -0.10 [-0.22, 0.03] | 93.9% |
|  | Superior | Abstinent | -0.12 [-0.25, 0.01] | 97.0% |
|  | Inferior | Non-Abstinent | -0.03 [-0.23, 0.18] | 61.4% |
|  | Mid-Transverse | Non-Abstinent | -0.04 [-0.23, 0.16] | 65.1% |
|  | Superior | Non-Abstinent | -0.05 [-0.24, 0.16] | 68.4% |

**Models 1-2 (Sleep and Group Main Effects).** Better last night sleep quality was associated with lower alpha power (*b* = -0.10, 95% CrI [-0.19, -0.01], PP = 97.6%) (Fig S1B). Group differences with respect to alpha power were identical to the primary results, given that this model was unchanged: the non-abstinent group demonstrated lower alpha power than the abstinent group (*b* = -0.36, [-0.82, 0.10], PP = 94.1%). Median alpha power was positive in the abstinent group (0.20, [-0.48, 0.14], PP = 87.4%) and negative in the non-abstinent group (-0.16, [-0.48, 0.14], PP = 86.5%). Fig S1C).


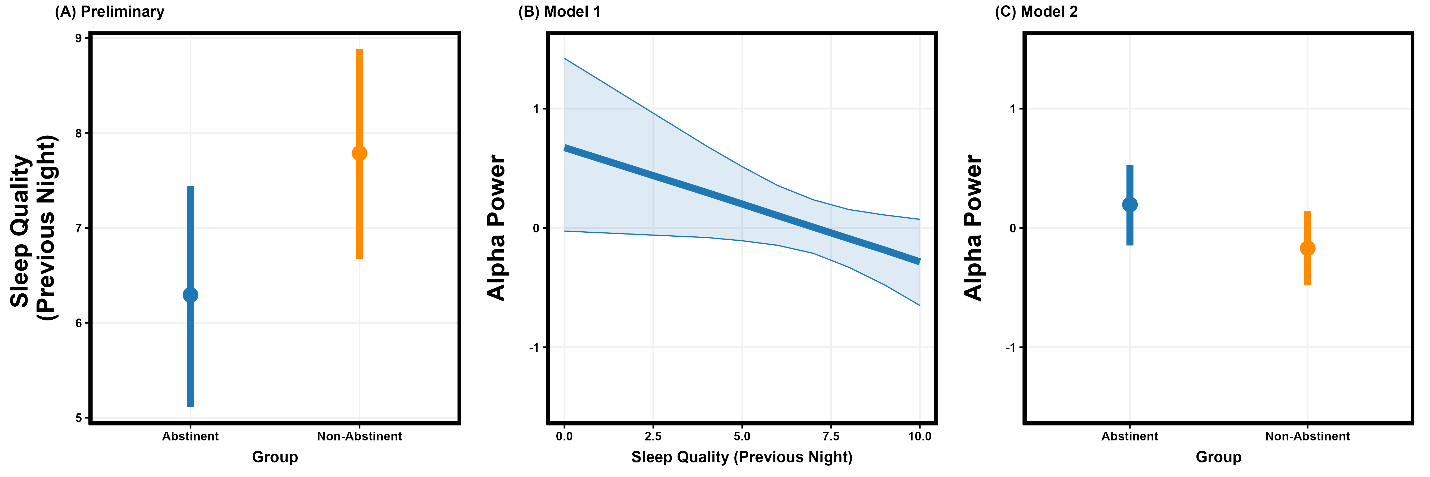
**Figure S1.** Preliminary Model and Main Effects (Models 1-2) for Previous Night. A) Sleep quality by group. B) Relationship between alpha power and sleep quality. C) Alpha power by group.

**Model 3 (Sleep x Group).** The effect of sleep on alpha power differed by group (sleep x group: *b* = 0.06, 95% CrI [-0.17, 0.30], PP = 70.1%). In the abstinent group, alpha power declined with higher sleep quality (slope = -0.10, [-0.22, 0.03], PP = 93.9%), while the non-abstinent group demonstrated no relationship between alpha power and sleep quality (slope = -0.04, [-0.23, 0.16], 64.2%), see Figure S2.

**
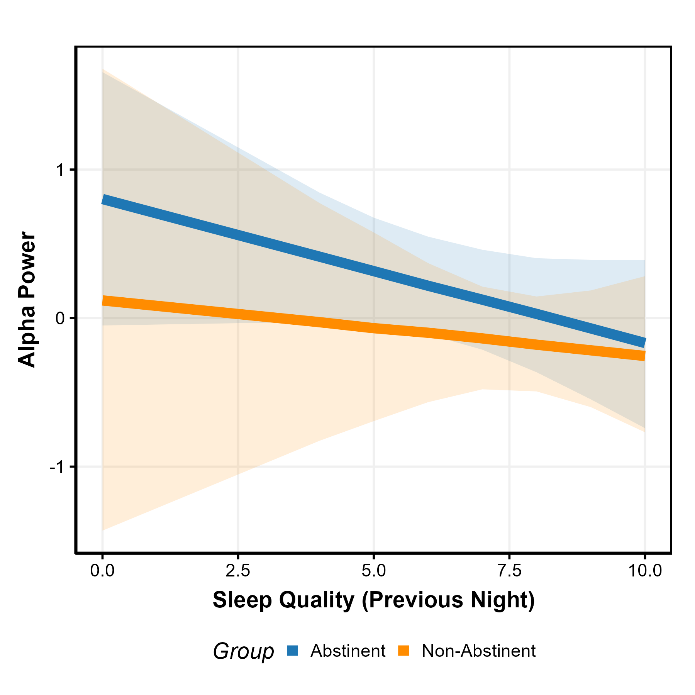
Figure S2.** Sleep by Group Interaction (Model 3) for Previous Night.

**Model 4 (Sleep x Group x Eyes).** The three-way interaction was supported (*b* = 0.08, 95% CrI [-0.04, 0.13], PP > 99.9%). As above, sleep quality was negatively related to alpha power in the abstinent group whether eyes were closed (slope = -0.09, [-0.21, 0.04], PP = 92.2%) or open (slope = -0.11, [-0.23, 0.02], PP = 94.9%). In the non-abstinent group, the association between sleep and alpha power was not supported when eyes were open (PP < 75%), but the association was negative when eyes were closed (slope = -0.07, [-0.27, 0.13]). Figure S3.


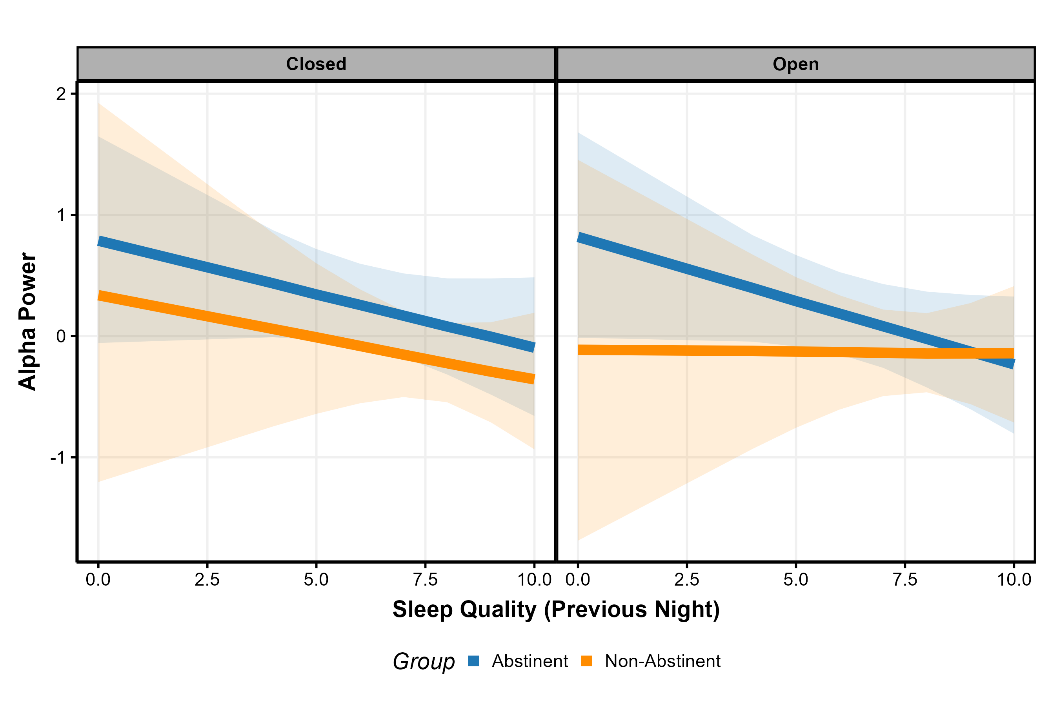
**Figure S3.** Sleep by Group by Eyes Interaction (Model 4) for Previous Night.

**Models 5-7 (Sleep x Group x Electrode Axis [ML/AP/IS]).** Each axis-specific three-way interaction was supported (ML: PP = 90.2%; AP: PP = 96.4%; IS: PP = 89.2%). For the non-abstinent group, sleep showed insufficient evidence for an association with alpha power across all regional strata (PP < 75). Conversely, sleep in the abstinent group exhibited strong to extreme evidence (PP = 95.6-99.9%) for an association with alpha power in every stratum, with effect sizes increasing when viewed from left to right (-0.07 to -0.13), anterior to posterior (-0.02 to -0.05), and inferior to superior (-0.03 to -0.05). Results are demonstrated in Figures S4-6.


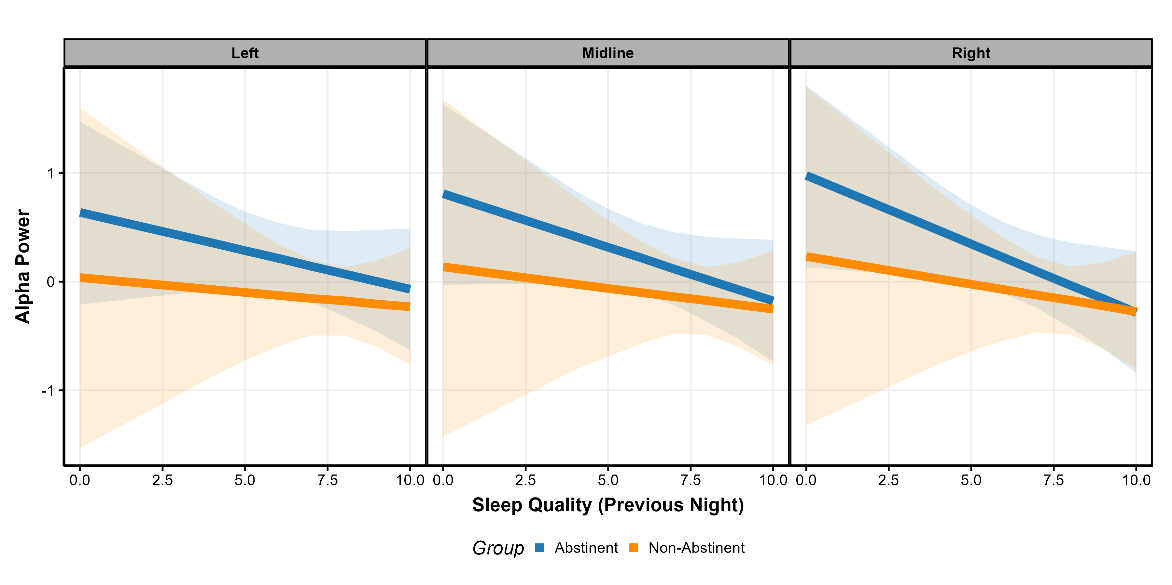
**Figure S4.** Alpha Power by Sagittal Plane for Previous Night.


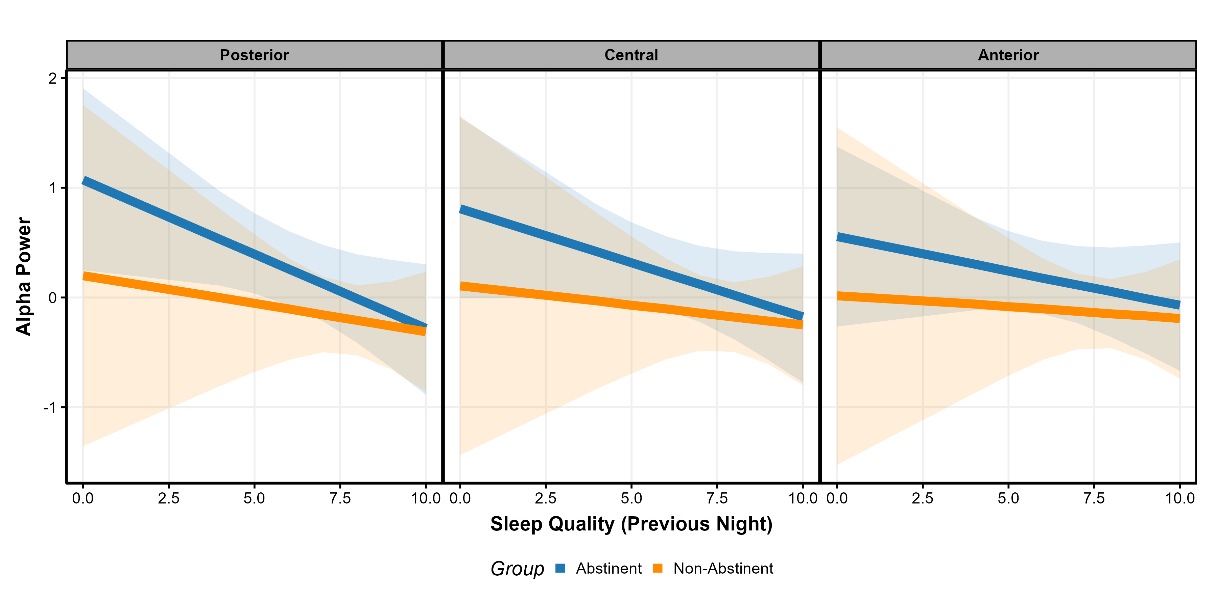
**Figure S5.** Alpha Power by Coronal Plane for Previous Night.


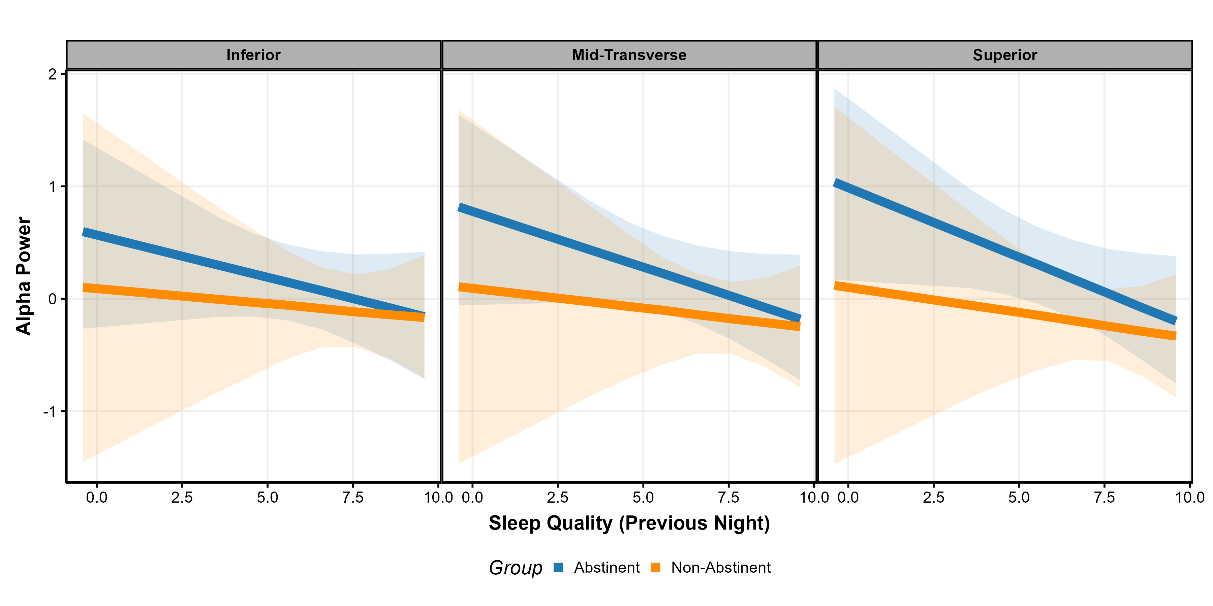
**Figure S6.** Alpha Power by Transverse Plane for Previous Night.

**Supplemental Figures from Manuscript**

The following three figures are supplemental to the main analyses presented in the main text of the manuscript. They demonstrate the effects of sleep and group on alpha power at the different electrode locations.


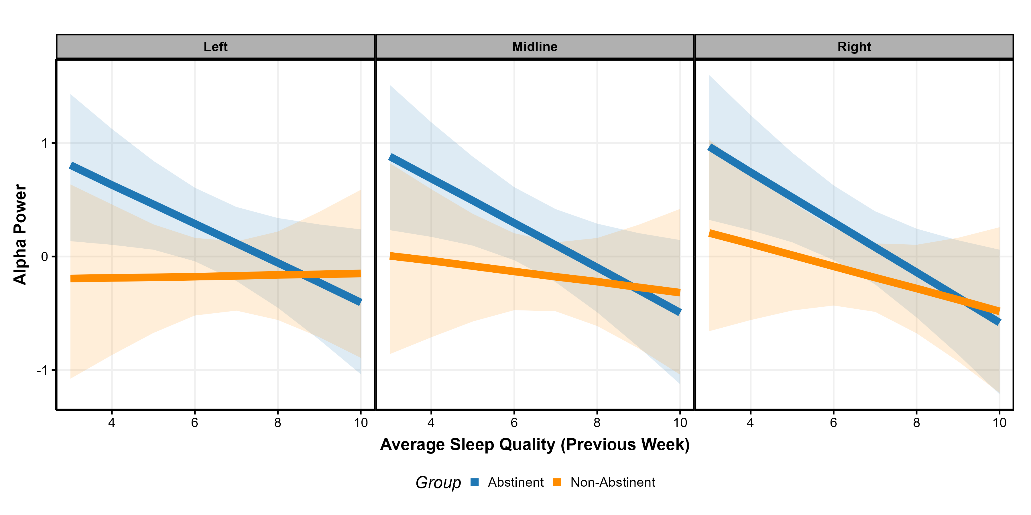
**Figure S7.** Alpha Power by Sagittal Plane for Previous Week. Effect sizes increased from left to right.


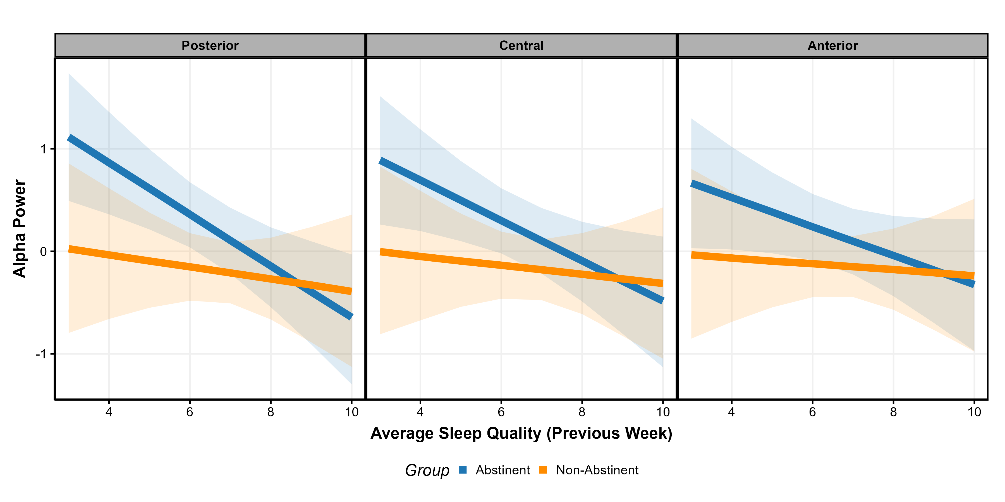
**Figure S8.** Alpha Power by Coronal Plane for Previous Week. Effect sizes increased anteriorly to posteriorly.


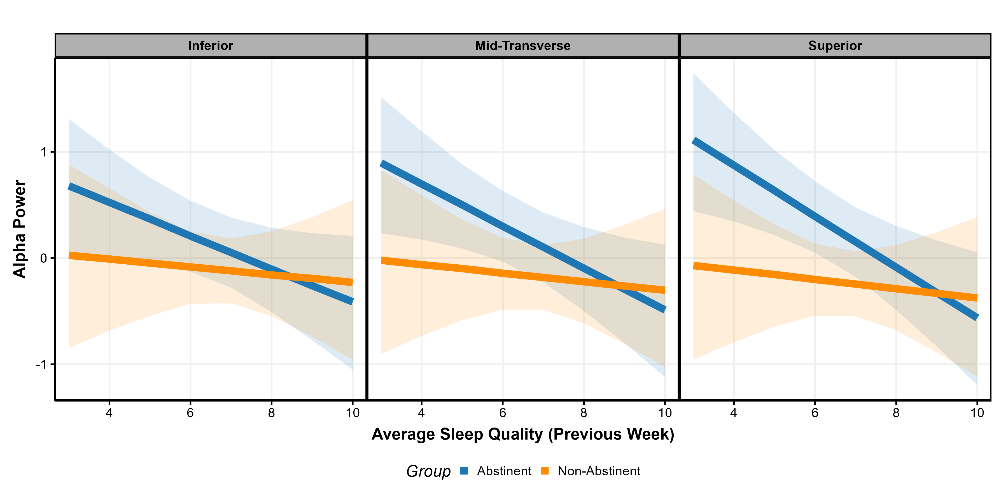
**Figure S9.** Alpha Power by Transverse Plane for Previous Week. Effect sizes increased inferiorly to superiorly.
